# Peering into Avian Influenza A(H5N8) for a Framework towards Pandemic Preparedness

**DOI:** 10.3390/v13112276

**Published:** 2021-11-15

**Authors:** Joshua Yi Yeo, Samuel Ken-En Gan

**Affiliations:** 1 Antibody & Product Development Lab, EDDC-BII, Agency for Science, Technology and Research (A*STAR), Singapore 138672, Singapore; samgan@apdskeg.com; 2 APD SKEG Pte Ltd., Singapore 439444, Singapore

**Keywords:** H5N8, influenza, virus, antiviral, mutation, reassortment, therapeutics, vaccines

## Abstract

2014 marked the first emergence of avian influenza A(H5N8) in Jeonbuk Province, South Korea, which then quickly spread worldwide. In the midst of the 2020–2021 H5N8 outbreak, it spread to domestic poultry and wild waterfowl shorebirds, leading to the first human infection in Astrakhan Oblast, Russia. Despite being clinically asymptomatic and without direct human-to-human transmission, the World Health Organization stressed the need for continued risk assessment given the nature of Influenza to reassort and generate novel strains. Given its promiscuity and easy cross to humans, the urgency to understand the mechanisms of possible species jumping to avert disastrous pandemics is increasing. Addressing the epidemiology of H5N8, its mechanisms of species jumping and its implications, mutational and reassortment libraries can potentially be built, allowing them to be tested on various models complemented with deep-sequencing and automation. With knowledge on mutational patterns, cellular pathways, drug resistance mechanisms and effects of host proteins, we can be better prepared against H5N8 and other influenza A viruses.

## 1. Influenza A Viruses and Its Subtypes

**Figure 1:**
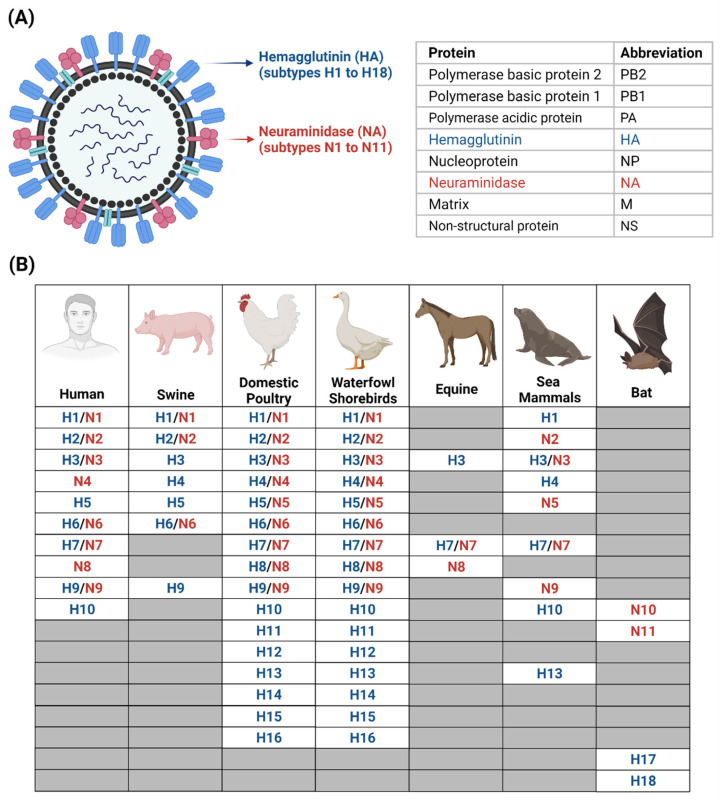
Influenza A viruses. (**A**) Structure of Influenza A viruses. (**B**) Hosts of Influenza A and their subtypes. Adapted from Mostafa et al. [6] and created with BioRender.com.

**Figure 2:**
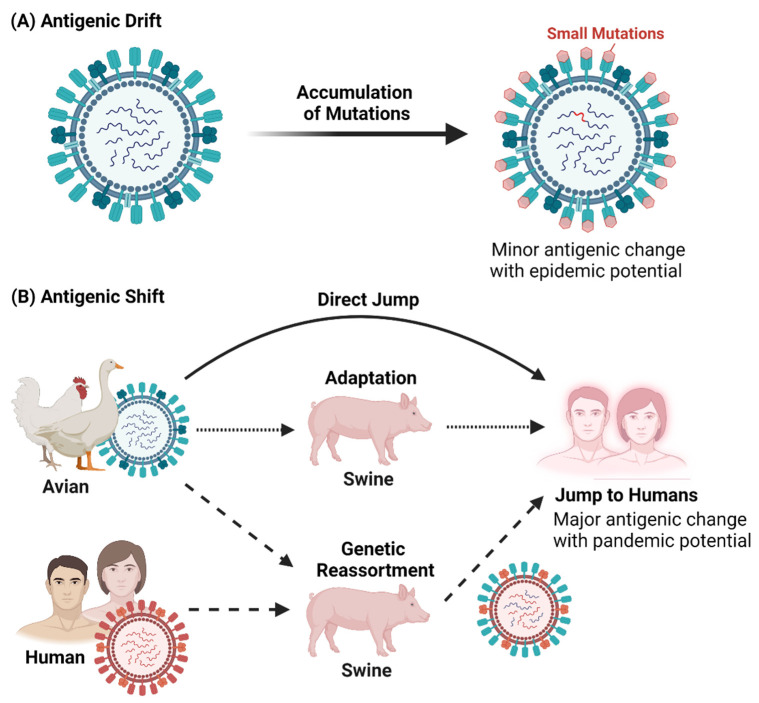
Antigenic Drift and Shift in Influenza A viruses. (**A**) Antigenic Drift results in minor antigenic changes from an accumulation of mutations. (**B**) Antigenic Shift results in major antigenic change via direct jump, adaptation and genetic reassortment. Created with BioRender.com.

Among the four influenza viruses (A, B, C and D), Influenza A viruses (IAVs) and Influenza B viruses (IBVs) have the potential to cause pandemics [1]. IAVs are divided into subtypes based on their viral surface glycoproteins: hemagglutinin (HA, subtypes H1 to H18) and neuraminidase (NA, subtypes N1 to N11). The Influenza A virion, studded with HA and NA, contains the viral genome that encodes for its proteins (see Figure 1A, [2]). The surface antigen, HA, binds to the terminal sialic acids of glycoprotein and glycolipids on host cells for viral entry, while NA cleaves the sialic acids to release the virions [3,4]. Given the importance of these viral proteins, their functions and the life-cycle of IAVs are extensively studied and reviewed [5,6,7,8].

IAVs are known to infect a broad range of hosts including humans, swine, domestic poultry, waterfowl shorebirds, equine, sea mammals and bats (see Figure 1B, [6,9]). The influenza A (H1N1) pdm09 virus that brought about the 2009 H1N1 pandemic resulted in the loss of an estimated 151,700 to 575,400 lives worldwide during its first year of circulation [10]. More recently, the first H5N8 human infection was reported in Russia on 18 February 2021 [11], reigniting interest on its transmission to humans and highlighting the importance of preparedness against H5N8 and similar influenza A viruses.

## 2. Epidemiology of H5N8

Named according to its HA 5 and NA 8 proteins, H5N8 first emerged on a farm in Jeonbuk Province, South Korea [12]. It was classified as a highly pathogenic avian influenza (HPAI) with an intravenous pathogenicity index (IVPI, the mean score per bird per observation over the 10-day period, ranging from 0 which indicates that no birds displayed clinical signs, to 3 which indicates the death of all birds [13]) of >1.2, the equivalence of ≥75% mortality [14]. First emerging in 2014, it spread throughout Asia, Europe, and the United States, infecting poultry and threatening public health, with this first wave subsiding in 2015. Attempts to control its spread led to the culling of more than 48 million poultry birds (the vast majority being chickens of which 38.4 million are egg-laying hens and 7.8 million turkeys) resulting in a loss of ~US$3.3 billion in the poultry industry [15] and further affecting food production. 

Fueled by the outbreak, myriads of viral sequences were deposited on the GISAID platform and catalyzed the initiation of The Global Consortium for H5N8 and Related Influenza Viruses [16,17]. One particular epidemiological study [17] brought into focus the vital role of long-distance migratory birds in the global spread of avian influenza viruses. The hemagglutinin (of clade 2.3.4.4) was also found to be remarkably promiscuous, capable of generating reassortants of multiple H5Nx IAVs of varying neuraminidase subtypes.

Shadowed by the COVID-19 pandemic, the recent 2020–2021 H5N8 outbreak affected both poultry and wild birds worldwide, particularly in Bulgaria, Czech Republic, Egypt, Germany, Hungary, Iraq, Japan, Kazakhstan, Netherlands, Poland, Romania, United Kingdom, and Russia [11]. In Astrakhan Oblast, Russia, an outbreak of clade 2.3.4.4b H5N8 caused the deaths of 11.2% of egg laying hens and the first H5N8 human transmissions to seven poultry farm workers and personnel (five females and two males of ages from 29 to 60 years old) on 18 February 2021 [11]. Despite being clinically asymptomatic with no transmission to close contacts, serological tests suggested recent infections. 

Through investigations using Madin-Darby Canine Kidney (MDCK) cells in a focus reduction neutralization assay (FRNA), four of the seven poultry workers who were PCR-positive had detectable FRNA titers in their initial serum samples [18]. Seroconversion was detected in their second serum samples fourteen days later, with one having a four-fold increase and another two having a two-fold increase in FRNA titer compared to their first serum sample. Their third serum sample, tested 44 days later, revealed a decrease in FRNA titers compared to their second serum samples. In the second and third serum samples, the maximum hemagglutination inhibition assay (HIA) titer observed was 1:20, and not indicative of zoonotic influenza infection. Using biolayer interferometry (BLI), specific IgG antibodies against influenza A/Astrakhan/3212/2020(H5N8) were detected for the five serum samples on the 14th day and for all samples on the 44th day.

While there was no evidence of human-to-human transmission of A/Astrakhan/3212/2020(H5N8) and its extended clade 2.3.4.4. viruses [19], the World Health Organization (WHO) continues to stress the importance of global surveillance and the need for continued risk assessment [11] to avert disastrous pandemics [20]. 

## 3. Understanding Transmission and Species Jumping

### 3.1. In Vitro, In Vivo and Ex Vivo H5N8 Models

To better illustrate the propensity of H5N8 to species jump, the potential of cross-species infection of H5Nx viruses have been extensively studied (summarized in Table 1). Evaluating the transmission and virulence of A/northern pintail/Washington/40964/2014(H5N2), A/gyrfalcon/Washington/41088-6/2014 (H5N8) and A/Thailand/16/2004(H5N1) [21], H5N2 and H5N8 were found to cause severe disease in high titers in mice. Similarly, in infected ferrets, efficient replication in the upper and lower respiratory tracts were associated with only mild clinical symptoms with no evidence of systemic infection. In fact, H5N8 could not be transmitted between ferrets through direct contact. Assessing viral replication kinetics in Calu-3 cells, H5N8 replication was found comparable to H1N1, but lower than H5N1. Interestingly, the replication of the H5Nx viruses were also significantly delayed and less efficient (especially during early replication cycles) at 33 °C than 37 °C.

Focusing on the pathogenesis of novel H5N8 isolates (A/broiler duck/Korea/Buan2/2014 and A/breeder duck/Korea/Gochang1/2014), both isolates did not result in mortality and notable respiratory symptoms in intranasally challenged ferrets [23], whereas intratracheally Buan2-infected ferrets exhibited dose-dependent mortality with no systemic infection. Analysis of the European A/Chicken/Netherlands/EMC-3/2014 (H5N8) showed low virulence with no detectable airborne transmission in ferrets [25]. Utilizing high-throughput RNA-sequencing to study differences between A/MD/Korea/W452/2014 (H5N8) and A/EM/Korea/W149/2006 (H5N1) viruses, viral transcripts and host immune-related genes expression levels were observed to be higher in H5N8-infected than H5N1-infected mice at 1-day post infection, but lower at 3- and 7-day post infection [26]. On the other hand, A/mallard duck/Korea/W452/2014(H5N8) was moderately pathogenic with limited tissue tropism in mice [24], inducing moderate levels of nasal wash titers from ferrets, being lethal and replicating systemically in chicken, attenuated but easily transmitted in ducks, with domesticated cats being more susceptible than dogs. Although A/mallard duck/Korea/W452/2014(H5N8) predominantly attaches to avian-like receptors, it can bind human virus-like receptors to replicate in human respiratory tract tissues.

The above studies on the various H5N8 strains show a consensus of moderate pathogenicity without severe disease in low doses for both in vivo mammalian models (mouse and ferrets) despite possible attachment to ex vivo human respiratory tissues with replication titers comparable to HPAI A(H5N1). It is with these findings that interesting insights to H5N8 can be made. 

### 3.2. Species Jumping from Avian to Humans

Influenza undergoes antigenic drift and shift [27,28,29] to change. The more minor changes in antigenic drift arise from host immune response evasion by the surface antigens HA and NA of IAVs (see Figure 2A, [29]). These initially small changes produce closely related IAVs, but can result in minor antigenically different viruses with epidemic potential through accumulation of mutations, keeping the host susceptible to IAVs due to such escape mutations.

Commonly resulting from genetic assortment, antigenic shift can occur when two antigenically distinct IAVs co-infect a common cell, allowing reassortment of their genome segments [29]. Such reassortments generate novel viruses of significantly different subtypes through new combinations of surface antigens (HA and/or HA and NA) from the pool of viral genes (see Figure 2B, [27]). The co-infection of an avian and human IAV can give rise to human-adapted viral polymerase antigens which the human host is immunologically naïve to, possibly occurring through an intermediate host such as, swine, which then transmits the human-adapted virus to humans [30,31]. In some cases, the species jump from avian to humans can occur directly [32].

While necessary for species jump, reassortment alone is unlikely to result in a human pandemic [29]. It is with the combined accumulation of mutations by the error-prone viral polymerase and selection of viruses that human-human aerosol transmission occurs, thus sowing the seeds of a pandemic. A total of 4 major adaptations from AIV to human-adapted viruses were identified [29,33]: (1) PB2 changes of 590/91SR, E627K and D701N; (2) Binding adaptation of HA α2-3 SA preferences to HA α2-6 SA; (3) Increased virion stability from high to low pH of fusion with increased heat stability; and (4) Evasion of restriction factors. Given that optimal AIVs growth temperature at the avian enteric tract is ~40–41 °C), the ability to replicate in the mammalian upper airway (32 °C) has been proposed as a mammalian adaptation marker [34,35]. With an increasing concern of viral receptor-binding adaptations, as illuminated by the COVID-19 pandemic, the receptor-binding adaptations of prior IAV pandemics and potential future adaptations have been extensively reviewed [36]. 

### 3.3. PB2 Subunit on Transmissibility and Virulence

**Figure 3:**
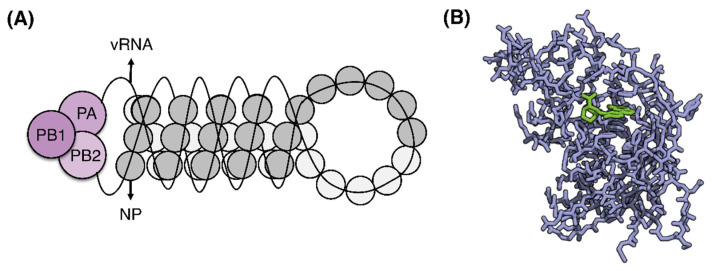
Polymerase basic protein 2 (PB2) subunit. (**A**) Ribonucleoprotein (RNP) complex comprising of the heterotrimeric complex (PB1, PB2, and PA), nucleoprotein (NP) and viral RNA (vRNA). (**B**) Influenza A (A/California/07/2009(H1N1)) PB2 complexed with Pimodivir, VX-787 (in green, PDB 7AS0 [42]) modified with QuteMol [46].

Apart from receptor-binding adaptations, the PB2 (polymerase basic protein 2) subunit is a major virulence and host transmission determinant [37,38,39,40,41], forming the ribonucleoprotein (RNP) complex with PB1 and PA (see Figure 3A). Due to its cap-snatching mechanism for mRNA transcription, the PB2 subunit has been proposed as a drug target (see Figure 3B, [42,43,44]). Primarily localized in the nucleus, the PB2 subunit can also accumulate in the mitochondria upon viral entry, interacting with the mitochondrial antiviral signaling protein (MAVS) while inhibiting MAVS-mediated beta interferon (IFN-β) expression [45]. Notably, only PB2 proteins of seasonal human influenza viruses associate with the mitochondria given their asparagine residue at amino acid residue 9 while the PB2 proteins of other avian influenzas viruses with aspartic acid do not [45].

The PB2-E627K substitution is a known mammalian signature mutation in IAVs. Deep-sequencing of H7N9 genomes showed genetic tuning of AIV in human hosts with the PB2-E627K substitution, suggesting association with H7N9 pathogenicity [47] and increased viral replication in mice [40,48,49,50,51]. Residues 283M and 526R of PB2 were found to synergistically contribute to virulence, with 283M established to be a mammalian-adapted virulence marker [52]. 

Although the precise mechanisms of these mutations remain unknown, three theories have been previously proposed [47]. Firstly, mutations such as E627K at the basic face of PB2 regulate polymerase activity and viral replication [53], possibly allows it to be catalytic active at the lower temperature of 33 °C in the human upper respiratory tract [54,55]. Secondly, through interactions of the PB2 C-terminus and cytoplasmic importins, PB2 can be independently imported into the nucleus prior to reconstitution of the polymerase unlike PA and PB1 [56]. This increases the charged surface residues (such as the E627K substitution) and thereby the association rate of PB2 to importins [57,58]. Thirdly, the mutations could influence interactions with different host factors, such as ANP32A [59], DEAD box RNA helicase DDX17/p72 [60], RIG-1 [61], and the Wnt/β-catenin signaling pathway [62].

## 4. Towards Pre-Emptive Therapeutics and Prophylactics

### 4.1. Reassortment and Mutational Studies

Genetic reassortment between avian H5N1 and human influenza viruses have been extensively investigated [63,64,65]. Coinfecting ferrets with both avian H5N1 (A/Thailand/16/04) and human H3N2 (A/Wyoming/3/03) viruses [63], continued exposure to H5N1 and seasonal influenza viruses was found to increase risk of generating H5 subtype reassortment viruses that can shed from upper airway secretions. Through reverse genetics, all 254 reassortants between avian H5N1 (A/chicken/South Kalimantan/UT6028/06) and human H3N2 (A/Tokyo/Ut-Sk-1/07) was generated [64], with the A/Tokyo/Ut-Sk-1/07 PB2 protein shown to allow efficient viral RNA transcription through its RNP activity. Furthermore, the reassortment of H5N1 viruses with human influenza viruses (H1N1, H3N2 and pandemic H1N1) in MDCK and human bronchial epithelial cells demonstrated that the neuraminidase and matrix genes of human influenza viruses had the highest genetic compatibility with H5N1 [65].

Certainly, the avian H5N8 virus has been shown to be capable of genetic reassortment with human influenza viruses (H3N2, H1N1 and pandemic H1N1) for viral titers and replication kinetics analysis in vitro using various cell lines [65] and in vivo using mice and ferret models [63,64]. Through simulating the genetic of avian H5N8 with other human influenza viruses in vitro reassortment (with emphasis on the PB2 subunit), it is possible to generate a predictive mutation platform like that performed for HIV [66], allowing us to understand their effects on viral replication and transmission,

As with many RNA viruses, Influenza A has low fidelity owing to its error-prone RNA-dependent RNA polymerase (RdRp) and lack of proofreading and repair mechanisms during genome replication [67,68,69]. This explains the mutation rates of A/Puerto Rico/8/1934 H1N1 and A/Hong Kong/4801/2014 (H3N2), which were found to be 1.8 × 10^−4^ and 2.5 × 10^−4^ substitutions/nucleotide/strand copied, respectively, with a transitional bias of 2.7–3.6 [70]. At a genomic level, this rate translates to an average of 2 to 3 mutations in each replicated genome, showing a relatively high amount during infection. Comprehensively mapping avian PB2 adaptation mutations [71], mutations with enhanced growth in human cells properties could be easily identified. Similarly, deep mutational scanning of the human A/Perth/16/2009(H3N2) hemagglutinin [72] can suggest mutational effects in the IAV antigenic drifts, possibly predicting strains heading towards lethal mutagenesis. Early methods to do these have been complicated by different escape mutations from polyclonal human immunity [73] confounding the analysis. Thereby, the use of an innate selection-free system [66] may provide a clearer insight into the influence of natural genetic code biases [74] to get a more accurate mutation rate of antigenic drift as for HIV [75] before in vitro co-infection of other IAVs to study Influenza reassortment.

### 4.2. Monitoring through Deep-Sequencing

The monitoring of emerging strains via deep-sequencing of viral genomes such as that performed on H7N9-infected clinical samples [47] can increase preparedness. PB2-M64T in the Danish novel clade 2.3.4.4b H5N8 viruses was found to be highly conserved in human Influenza A H1N1, H2N2, H3N2 viruses [76,77], in A/barnacle goose/Denmark/14139-3/2020(H5N8) [78] and A/chicken/Netherlands/20017694-004/2020(H5N8), but not the recent first human infection, A/Astrakhan/3212/2020(H5N8). Through close monitoring, the sequences were mapped to anticipate species jumping [76] from comparing 42 previously identified human-adaptive markers of PB2 sequences.

Following-up on the first human infection of A/Astrakhan/3212/2020(H5N8), whole genome sequence and virus characterization of the human influenza isolate A/Astrakhan/3212/2020(H5N8) and five avian isolates (A/chicken/Astrakhan/321-01/2020, A/chicken/Astrakhan/321-05/2020, A/chicken/Astrakhan/321-06/2020, A/chicken/Astrakhan/321-09/2020, A/chicken/Astrakhan/321-10/2020) [18] were phylogenetically determined. The HA and NA genes of the human isolate were found to be identical to the avian isolate A/chicken/Astrakhan/321-06/2020(H5N8), with a S28N mutation in the NA that was not found in the other four avian isolates. Mutations at this 28N site is also present in candidate vaccine viruses but this could be due to a methodological bias introduced by nested PCR [79]. At the HA, A/Astrakhan/3212/2020(H5N8) also had the polybasic proteolytic cleavage site (PLREKRRKR/G), confirming its HPAI virus identity. At the polymerase acidic protein (PA) gene, the human isolate showed the A598T distinction from the avian isolates that while currently having an unknown impact, provides a clue to an important gap on the species differences, not only of the mutation but of the NA gene function differentially in different hosts.

Compared to its closest antigenic reference strain of clade 2.3.4.4. b A/Fujian-Sanyuan/21099/2017, the A/Astrakhan/3212/2020 had the T140A substitution in antigenic site A, likely associated with antigenic drift [18]. Both the A/Fujian-Sanyuan/21099/2017 and A/Astrakhan/3212/2020 strains had the same receptor-binding site (RBS) markers and a QS(R)G motif at the RBS associated with an avian-like α2,3-sialic acid receptor-binding preference [80]. Genotypic analysis of A/Astrakhan/3212/2020 also revealed that it did not have mutations associated with reduced susceptibility to NA inhibitors, adamantanes or baloxavir marboxil [81], while phenotypic analysis of both human and avian isolates demonstrated normal susceptibility to oseltamivir and zanamivir.

### 4.3. Contribution of Host Proteins

**Figure 4:**
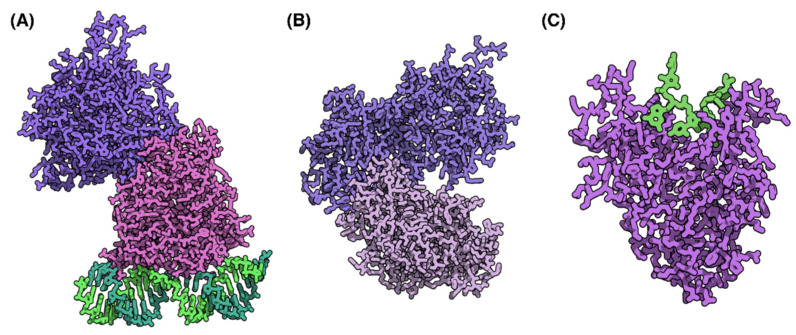
Host Proteins with RNA editing functions. (**A**) Human Adenosine Deaminase Acting on dsRNA (in purple) bound to dsRNA (in green, PDB 5ED1, [83]). (**B**) Apolipoprotein B mRNA-editing enzyme, catalytic polypeptide-like 3G (APOBEC3G, PDB 6P3X, [82]). (**C**) Zinc-finger antiviral protein (ZAP) bound to RNA (in green, PDB 6L1W, [93]). Protein structures modified with QuteMol [46].

RNA editing by host proteins in higher eukaryotes can occur, such as those by adenosine deaminases acting on RNA (ADAR) and apolipoprotein B mRNA-editing enzyme catalytic polypeptide (APOBEC), which deaminates adenine (A) to inosine (I), recognized as guanosine (G), and cytidine (C) to uracil (U), recognized as thymine (T) respectively (see Figure 4A,B, [82,83,84,85,86,87]). A previous study involving H1N1, H3N2, H5N1 and H7N9 in both human (lung and tracheobronchial cells) and avian (ileum and lung tissues) hosts [88] showed strong induction of APOBEC3G but not APOBEC3F by influenza A. This upregulation of APOBEC3G was attributed to the IFN-β response, although it did not translate to antiviral activity [89]. Thus, it is of value to understand how the mutation rate and mutational bias influenced by these host proteins can contribute to H5N8 viral proteins species jump and inhibition of viral infections [90]. The inclusion of zinc-finger antiviral protein (ZAP, see Figure 4C), given its ability to recognize RNA and antiviral activity of diverse RNA viruses (including IAV) through depleting vRNAs with high frequencies of CG dinucleotides [91,92,93,94] may also provide deeper host-viral interactions. Aside from the discussed host proteins, understanding restriction factors and their mechanism of action are key to the development of therapeutics and prophylactics [95], as exemplified by the autophagy regulator TBC1D5 which controls IAV replication and promote lysosomal targeting of its M2 protein [96] and a natural variant (D130A) in ANP32B which impairs dimeric influenza virus polymerase formation and viral replication [97].

### 4.4. Design-Build-Test-Learn Cycle for H5N8

**Figure 5:**
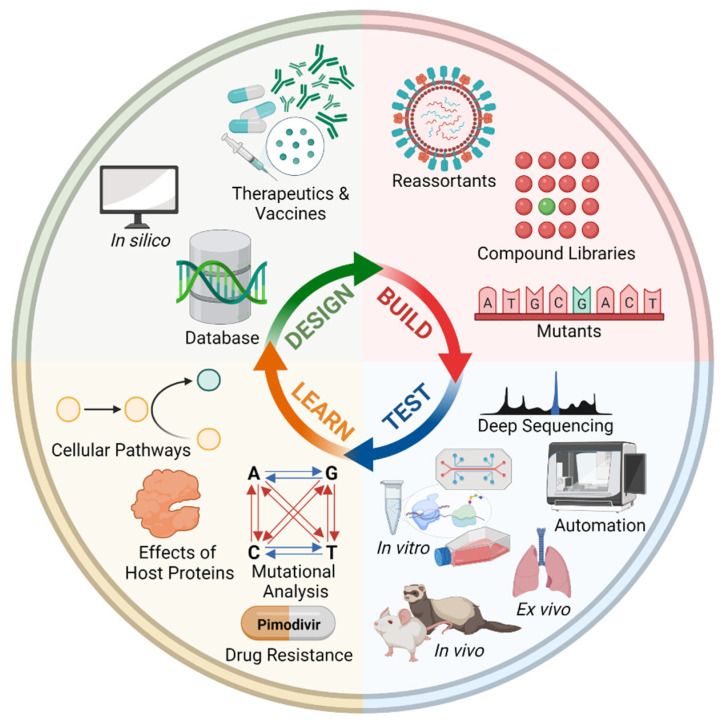
Framework of a design-build-test-learn (DBTL) for H5N8 to complement surveillance. Created with BioRender.com.

Learning and adapting from the design-build-test-learn (DBTL) cycle commonly utilized in microbial engineering [98,99], a framework (exemplified in Figure 5) can guide therapeutics and prophylactics development [100]. This has been previously proposed and extensively reviewed for the identification and production of novel active flavonoids against the main protease of SARS-CoV-2 [101] and more recently, towards building a sustainable vaccines industry away from conventional approaches through designing and prototyping of vaccines in biofoundries [102].

Through an understanding of H5N8, there is transferable understanding and insight for other influenza A viruses such as the first avian influenza A(H10N3) human infection in Zhenjiang City, Jiangsu Province, China, reported in May 2021 [103,104]. Libraries of possible mutations, reassortants and compounds can be combinatorically and simultaneously tested and validated through in vitro, in vivo and ex vivo experimentations (as listed in Table 1). Aside from models previously discussed, a human lung airway-on-a-chip model to study emerging influenza virus variants has been recently established, demonstrating the emergence of clinically associated drug resistance mutations in the presence of antiviral drugs [105]. Through rational experimental designing, this developmental process can be automated. Additionally, given the advances in sequencing technologies, a previously built pipeline has been shown to discriminate clonotypes of IAV genes using the MinION platform [106], with implications on advancing viral quasispecies investigations [107] and as part of global surveillance. Considering host proteins and restriction factors, predictions of mutations (as previously applied to HIV-1 [66]) can provide insights to emerging drug resistance (of available and novel drugs) and cellular pathways. It should be noted that there would be two aspects to such experimental mutational platform for Influenza. In studying the innate RdRp bias and hotspots in antigenic drift, the method applied for HIV would suffice. However, for studying antigenic shift, in vitro co-infection with other IAV subtypes would need to be performed with a screening for interactions with anti-serums or with other interventions to study escape variants. For the latter, there is much to do, perhaps best and most easily performed at an in silico level for cost-effectiveness and safety reasons. Moreover, such computational analysis can also support intervention strategies.

With proteomics data of IAVs [108,109], novel drug targets and drug repurposing strategies can be identified as previously applied to SARS-CoV-2 [110]. Taking a host-directed therapy approach [111], the constructed SARS-CoV-2-induced protein artificial neural network cross-examined disease signatures and approved drugs, identifying 200 drugs with 40 already in clinical trials and 2 (proguanil and sulfasalazine) demonstrated to inhibit replication [110]. Applying such an approach to the H5N8 subtype and extended IAVs, understanding these metabolic perturbations are key in identifying novel and repurposing existing drugs to target them [112]. Some of these cellular pathways that are hijacked during influenza infections include the metabolic pathways and intracellular signaling cascades NF-κB, PI3K/Akt, MAPK, PKC/PKR and TLR/RIG-I [112,113].

With feedback from the already huge databases available for influenza viruses [16,114,115,116,117,118], the framework can leverage upon in silico and machine learning prediction methods. One example is to utilize the stacking model to differentiate mutation patterns and antigenicity between epidemic and pandemic strains for influenza surveillance, as was applied to H1N1 viruses [119]. Sequence-only fitness estimates, applied to the seasonal H3N2 influenza virus, could also allow a forecasting framework integrating estimates of phenotypic measures of antigenic drift and functional constraint to be built [120], amongst the many available methods. An example is Tempel, a time-series mutation prediction model for influenza A viruses employed recurrent neural networks with attention mechanisms for historical glycoprotein hemagglutinin sequences to predict mutations likely to occur in flu seasons [121].

Combined, the framework guides the development of antiviral therapeutics and vaccines, that includes small molecules [122,123,124], antibodies, and universal influenza vaccines [125]. One example of such rational drug designs is JNJ7918, an oral small molecule mimicking broadly neutralizing antibodies, that was improved for binding and virus neutralization, and further refined for stability and oral bioavailability [124]. Another oral inhibitor is Pimodivir (VH-787, Figure 3B), a novel inhibitor of influenza virus replication, inhibits cap binding to the PB2 subunit [126] and recently entered the third phase of clinical trials [42]. However, several major drug-mutations in the PB2 subunit has already been observed (such as F404Y and M431I and H357N, [42]) to show resistance. Thus, the mapping of PB2 single-amino-mutations could prepare against Pimodivir resistance [127]. For it is through predicting and understanding mutations that future inhibitors (or improvement on existing inhibitors such as Pimodivir) which could possibly withstand drug-resistance mutations [127] be better designed. This combinatorial approach of computational and experimental research has been previously applied in the COVID-19 pandemic, for both drug repurposing [128] and synergistic drug combinations [129]. Such knowledge, when coupled with antibody engineering [130], could also lead to development of better neutralizing antibodies, particularly if they are of mucosal antibodies of IgA [131] or even IgE [132,133], capable of recognizing superantigen elements [134] as the SARS-CoV-2 spike [135].

Since escape mutations also impact vaccines, the assessment of safety and immunogenicity is also important, as was performed for the first-in-human universal flu vaccine Phase I trial of FluMos-v1 [136], which is a quadrivalent influenza nanoparticle vaccine containing 20 HA glycoprotein trimers that induced broad protection in mice, ferrets and monkeys [137].

Given that nucleoside analogues can exhibit a broad-spectrum antiviral effect in lethal mutagenesis [138,139] by augmenting mutation rates towards error catastrophe [140], the trajectory and mutational patterns of the viral mutation have clear implication in unraveling such effects. Three nucleoside analogues, ribavirin, 5-azacytidine and 5-fluorouracil active against seasonal H3N2 (A/Panama/2007/1999(H3N2) and A/Wyoming/03/2003(H3N2)) and laboratory-adapted H1N1 (A/Puerto Rico/8/1934(H1N1) and A/WSN/33(H1N1)), promoted the increase in defective viral particles [141]. The testing of these nucleoside analogues against H5N8 viruses in the framework could allow assessment of their use as a broad-spectrum anti-influenza drug. With its patent pending, a novel class of immunostimulatory RNAs (isRNAs) simulating Type I Interferon (IFN-1) response [142] inhibited infection of multiple respiratory viruses (including SARS-CoV-2, influenza, and common cold viruses) with >95% influenza inhibition and >99% SARS-CoV-2 inhibition in vitro in human lung epithelial cells, and inhibition of SARS-CoV-2 infection in vivo in hamsters.

**Figure 6:**
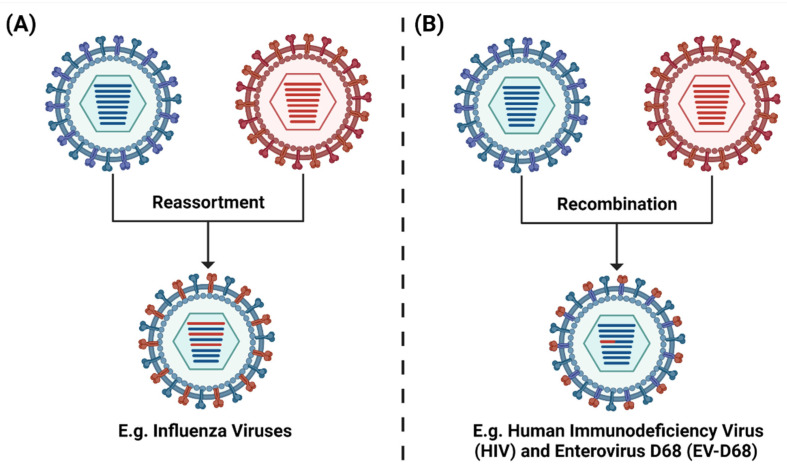
Modes of genetic modification by viruses. (**A**) Reassortment, which only occurs in segmented RNA viruses such as Influenza viruses where two antigenically distinct IAVs co-infect a common cell and reassort their genome segments. (**B**) Recombination, which occurs in all RNA viruses such as Human Immunodeficiency Virus (HIV) and Enterovirus D68 (EV-D68). Created with BioRender.com.

Since different viruses have different mutational methods and rates, where reassortment occurs in segmented RNA viruses and recombination for all RNA viruses ([143], see Figure 6), there is room for cross-application and cross-validation of the computational and experimental prediction methods. By adapting this framework to substitute reassortants with recombinants, it can potentially be applied to other viruses which undergo recombination such as Human Immunodeficiency Viruses (HIV) [144,145] and Enterovirus D68 (EV-D68) [146].

## 5. Conclusions

With the first human transmission of influenza H5N8 in Astrakhan Oblast, Russia, the need to understand this emerging highly pathogenic avian virus and its possible mechanisms of species jumping to avert disastrous pandemics is demonstrated. Through the incorporation of novel methods in a design-build-test-learn (DBTL) cycle, gaps in understanding Influenza and other viruses can be made through models complemented with deep sequencing and automation. In combination with in silico prediction methods, this knowledge can be applied towards development of antiviral therapeutics and vaccines.

## Figures and Tables

**Table 1:** Studies on H5Nx viruses and their utilized models.

Virus Strain	Model	Pathology	Study
A/environment/Hong Kong/WCRB-01/2018(H5N6); A/spoonbill/HK/17-18259/2017(H5N6); A/northern pintail/HK/MP692/2016(H5N6); A/chicken/Egypt/F1366A/2017(H5N8); A/grey-headed gull/Uganda/200144/2017(H5N8)	Human Airway Organoids and Alveolar Epithelial Cells	Replicated productively with similar virus titers; Lower virus titers than human isolates A(H1N1)pdm09, HPAI A(H5N1) and HPAI A(H5N6); Differential cellular tropism; Induced low levels of pro-inflammatory cytokines and chemokines; Zoonotic potential but low transmissibility among humans	[22]
A/northern pintail/Washington/40964/2014(H5N2); A/gyrfalcon/Washington/41088-6/2014(H5N8)	Mice	Cause severe disease at high doses	[21]
	Ferret	Efficient replication in upper and lower respiratory tracts; Mild clinical symptom; No systemic infection	
	Calu-3 Cells	Replication levels lower than virulent H5N1 but comparable to human seasonal virus	
A/broiler duck/Korea/Buan2/2014(H5N8); A/breeder duck/Korea/Gochang1/2014(H5N8)	Ferret	Low pathogenesis against ferrets; No systemic infection; Both isolates did not induce morality and significant respiratory signs when intranasally challenged; Buan2-infected ferrets demonstrated dose-dependent mortality when intratracheally challenged	[23]
A/mallard duck/Korea/W452/2014(H5N8)	Mice	Moderately pathogenic; Replicated moderately in lungs; Limited tissue tropism, particularly brain tissues; Less pathogenic than H5N1 isolates; When inoculated intranasally, resulted in body weight reduction of 6% and 40% lethality within 14 days	[24]
	Ferret	Induced moderate nasal wash titers; Shed from upper respiratory tract; Replicated in lungs and spleen, recovered from brain, liver and intestine; Transiently evaluated body temperature without notable signs of illness when intranasally inoculated	
	Chicken	Highly pathogenic, exhibiting disease signs; Lethal; Replicated systemically	
	Duck	Severe-to-moderate signs of infection; Attenuated; Efficiently transmitted; 17% succumbed to infection when oronasally infected; High levels of virus replication in lungs, hearts and intestines are compared to oropharynx and cloaca; Not found in brain tissue samples	
	Dogs	No efficient replication in upper nasal cavity and visceral tissues; No observable signs of illness	
	Cats	Transient fever; Marginal weight loss	
	MDCK Cells	Form smaller plaques than H5N1 isolates; Spherical virus particles under transmission electron microscopy	
	SPF Eggs	Grows more rapidly than H5N1 isolate with exception of En/W149(H5N1) with PB2_627K_ mutation	
	Differentiated Primary CELu	Lower replication titers than En/W149(H5N1)	
	NHBE		
	Human Nasal Respiratory Epithelium and Lung Tissues	Replication titers comparable to HPAI A(H5N1); Attachment to human respiratory tissues	
A/Chicken/Netherlands/EMC-3/2014(H5N8)	Ferret	Replicates poorly; Did not develop severe disease or clinical signs; Lacks ability to transmit airborne	[25]
A/MD/Korea/W452/2014 (H5N8); A/EM/Korea/W149/2006 (H5N1)	Mice	Higher viral transcript and host immune-related genes expression in H5N8-infected compared to H5N1-infected mice at 1-day post infection; Lower number of H5N8 genes at 3- and 7-day post infection than H5N1	[26]

## Data Availability

No new data were created or analyzed in this study. Data sharing is not applicable to this article.
